# Ni^2+^‐Directed Anisotropic Growth of PtCu Nested Skeleton Cubes Boosting Electroreduction of Oxygen

**DOI:** 10.1002/advs.202104927

**Published:** 2022-03-10

**Authors:** Yafeng Zhang, Kai Ye, Qianru Liu, Juan Qin, Qike Jiang, Bing Yang, Feng Yin

**Affiliations:** ^1^ School of Physics and Information Technology Shaanxi Normal University Xi'an 710119 China; ^2^ Dalian National Laboratory for Clean Energy Dalian Institute of Chemical Physics Dalian 116023 China; ^3^ CAS Key Laboratory of Science and Technology on Applied Catalysis Dalian National Laboratory for Clean Energy Dalian Institute of Chemical Physics Dalian 116023 China; ^4^ School of Physics and Information Technology Key Laboratory of Syngas Conversion of Shaanxi Province Shaanxi Normal University Xi'an 710119 China

**Keywords:** nested skeleton cubes, octahedral stars, anisotropic growth, PtCu electrocatalysts, oxygen reduction reaction

## Abstract

Structure‐controlled Pt‐based nanocrystals have the great potential to provide a flexible strategy for improving the catalysis of the oxygen reduction reaction (ORR). Here, a new synthetic approach is developed to tune the 3D structure of Pt‐based alloys, and switch a synthetic reaction which produces solid PtCu octahedral stars (OSs) to produce PtCu nested skeleton cubes (NSCs) by simple addition of Ni(acac)_2_. In particular, Ni^2+^‐guided anisotropic growth is observed to generate the nested skeleton structure in PtCu NSCs. Ni^2+^, though absent from the nanoalloys, not only endows faster Cu reduction kinetics but also acts as a structure‐directing agent. Moreover, it is shown that acetic acid treatment of PtCu NSCs/C exposes Pt‐rich surface with a fine‐tuned Pt d‐band center energy and the reduced Cu leaching, resulting in strikingly high activity and stability. Acid‐treated PtCu NSCs/C shows a remarkable ORR mass activity of 5.13 A mg_Pt_^–1^, about 26 times higher than commercial Pt/C catalyst. This catalyst also exhibits excellent stability with a lower activity decay of 11.5% and the negligible variation in structure after 10 000 cycles.

## Graphical abstract

An effective strategy is proposed to tune the 3D structure of Pt‐based alloys, and switches a synthetic reaction which produces solid PtCu octahedral stars (OSs) to produce PtCu nested skeleton cubes (NSCs) by simple addition of Ni(acac)_2_. An enhanced oxygen reduction reaction (ORR) activity is obtained on PtCu NSC catalysts, which is higher than that of the solid PtCu OS catalysts.

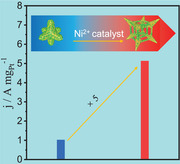

## 1 Introduction

The high cost and limited availability of commercial Pt catalyst for the cathodic oxygen reduction reaction (ORR) is a bottleneck for the production and use of various types of fuel cells.^[^ ^1^ ^]^ The ability to synthesize low cost, high‐activity, and high‐stability catalysts would be a considerable advance in fuel cell technology and Pt‐based alloys have widely been considered promising candidates in this area.^[^ ^2^, ^3^, ^4^, ^5^ ^]^ The structure and composition of such Pt‐alloys play a very important role in determining their catalytic properties and there has been considerable progress in the tuneability of 3D structure and composition of Pt‐alloys, including the production of solid, hollow, and skeleton structures.^[^ ^6^, ^7^, ^8^, ^9^, ^10^, ^11^, ^12^, ^13^ ^]^ Skeleton structures have attracted particular interest for their highly open 3D structures.^[^ ^14^, ^15^, ^16^ ^]^ The accessibility of 3D alloy surfaces in skeleton structures to reactant O_2_ molecules has been documented to increase the specific activity for ORR.^[^ ^9^, ^10^ ^]^ In addition, these nanoscale skeletons are made up of interconnected ultrafine 1D nanostructures, which greatly increases the electrochemical active surface area (ECSA) of the catalyst.^[^ ^11^, ^17^ ^]^ Studies on alloy catalysts have also shown that ORR‐favorable activity and stability could be improved by a Pt‐rich surface.^[^ ^9^, ^12^, ^18^, ^19^ ^]^ For example, in the work on bunched PtNi alloy nanocages by Baoyu Xia et al.^[^ ^18^ ^]^ the stable catalyst modified to give a Pt‐rich surface showed an ORR mass activity of up to 17 times higher as compared to Pt/C. All of these effects have been explored to create substantial improvement in the mass activity of Pt in ORR.^[^ ^9^ ^]^ However, the nanoframe field has mostly been focused on the synthesis and formation mechanism of single‐layered Pt‐alloy skeletons.^[^ ^14^, ^16^ ^]^ In this work, we show a new method to tune the 3D structure of Pt‐based alloys in synthesis and generate PtCu nested skeleton cubes (NSCs) by simple addition of Ni(acac)_2_. An enhanced ORR activity was obtained on PtCu NSC catalysts, which was far better than that of the solid PtCu octahedral star‐shaped catalysts generated by the synthesis without Ni(acac)_2_ and commercially available Pt/C catalyst. Moreover, surface treatment of PtCu NSCs/C catalyst by acetic acid exposed a Pt‐rich surface, leading to a 25.7‐fold enhancement in mass activity compared to Pt/C catalyst. In addition, this catalyst presented a dramatic increase in stability in ORR even after 10 000 reaction cycles compared to commercially available Pt/C and other PtCu alloy catalysts.

## 2 Results and Discussion

### 2.1 Synthesis and Structures of PtCu OSs/NSCs

**Figure 1:**
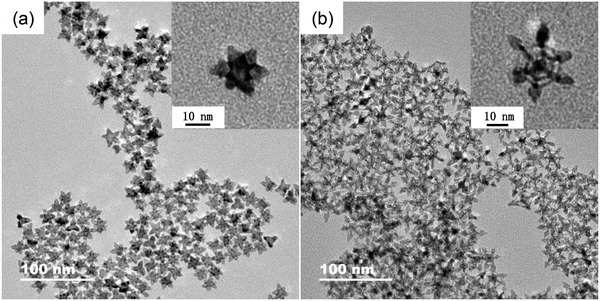
TEM images of a) PtCu OSs and b) PtCu NSCs.

PtCu nanocrystals were prepared in an oleylamine (OAm) solution involving Cu(acac)_2_, Pt(acac)_2_, and cetyltrimethyl ammonium bromide (CTAB) molecules at 175 ℃ for 8 h with and without the presence of Ni(acac)_2_. When the reaction was performed without Ni(acac)_2_, transmission electron microscope (TEM) imaging showed nanostructures resembling six‐pointed stars of roughly uniform size (**Figure** **1**a). In the presence of Ni(acac)_2_, the nanostructures obtained were of a notably different shape: TEM image showed hexagonal shapes also of a roughly uniform size with a sharp spike at every corner (Figure 1b). Upon closer inspection of TEM images, the six‐pointed stars appeared to be solid, while the hexagonal structures appeared to be skeletal. To determine the 3D structure, both samples were rotated and TEM images of an individual nanocrystal were collected at −20°, 0°, 20°, and 45° (−35°, −15°, 0°, and 25°, see Figures S1 and S2, Supporting Information). By comparing 2D projections of proposed 3D model structures with TEM images, the apparently star‐shaped nanocrystals were identified as solid octahedral stars (OSs, see Figure S1 in the Supporting Information), while the hexagonal nanocrystals were identified as nested skeleton cubes (NSCs) with spikes at their vertices (Figure S2, Supporting Information).

**Figure 2:**
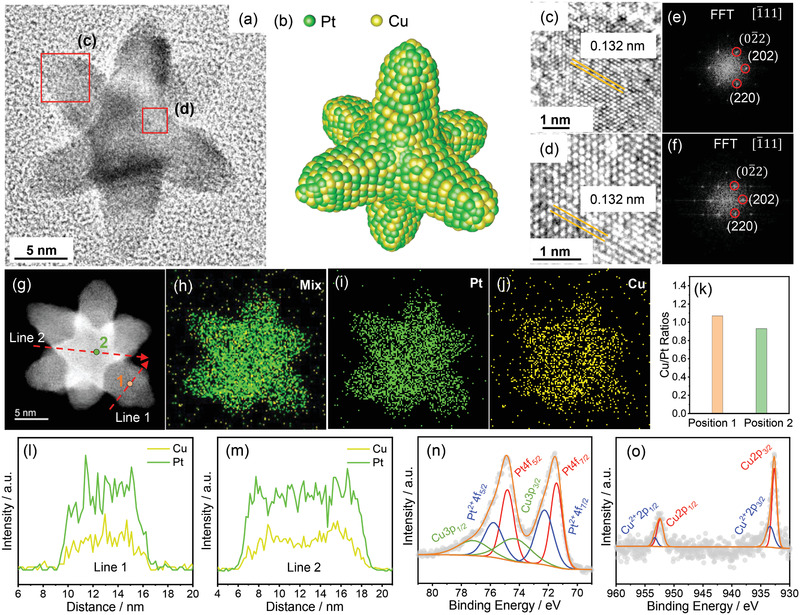
a) HRTEM image of single acid‐treated solid PtCu OS. b) Corresponding model schematic illustration. c,d) Enlarged HRTEM images of the selected areas shown in (a). e,f) Corresponding fast Fourier transform (FFT) patterns. g) STEM image of single acid‐treated solid PtCu OS. h–j) Corresponding EDS elemental mappings. k) EDS elemental ratios of specific positions in (g). l,m) EDS line scan profiles along red dotted arrow directions in (g). n) Elemental Pt, and o) Cu XPS spectra for PtCu A‐OSs/C.

For characterization of the precise structures of these two types of nanocrystals (OSs and NSCs), the nanocrystals were treated with acetic acid to remove surface species including organics and oxides. High resolution TEM (HRTEM) image of a single acid‐treated OS shows a solid star‐shaped structure similar to the untreated nanocrystal (**Figure** **2**a). Locally amplified HRTEM images of both the star‐shaped arms and the central region of the nanocrystal (marked with red‐squares in Figure 2a) show well‐resolved interplanar spacings of 0.132 nm (Figure 2c,d). Both the interplanar spacing and Fourier transforms of the HRTEM images are consistent with those of the terminated‐{220} facets of face center cubic (fcc) PtCu alloys in both regions of the nanocrystal (Figure 2c–f). Furthermore, X‐ray diffraction (XRD) experiment performed on acid‐treated PtCu OSs/C (PtCu A‐OSs/C, see Figures S3a and Figure S4 in the Supporting Information) also verifies a fcc structure similar to that of fcc Pt_50_Cu_50_ alloys (PDF#48‐1549). The composition of the nanocrystal was probed using energy dispersive X‐ray spectroscopy (EDS) analysis: the overall atomic ratio of Cu/Pt in PtCu A‐OSs/C is measured to be 48.7/51.3 (Figure S5, Supporting Information), which is consistent with the result of the inductively coupled plasma optical emission spectrometry (ICP‐OES) analysis. Only a small amount of Cu (roughly 4%) is lost after acetic acid treatment due to surface oxide leaching. Scanning TEM‐EDS (STEM‐EDS) elemental mappings (Figure 2g–j) show that the Pt (green) and Cu (yellow) are well‐mixed and evenly distributed throughout the OS and this is also confirmed by EDS line scan analyses across central and arm regions (Figure 2l,m). The measured atomic Cu/Pt ratio in the arms (position 1 in Figure 2g,k) and the central region (position 2 in Figure 2g,k) of the star is very close in value: 1.07 and 0.93, respectively. The chemical states of various components in PtCu OSs were confirmed by X‐ray photoelectron spectroscopy (XPS) with both zero‐valent Pt and Cu dominating in PtCu OSs before and after the acid treatment (Figure 2n,o; Figure S6, Supporting Information),^[^ ^20^ ^]^ confirming that the nanocrystal is a PtCu alloy.

**Figure 3:**
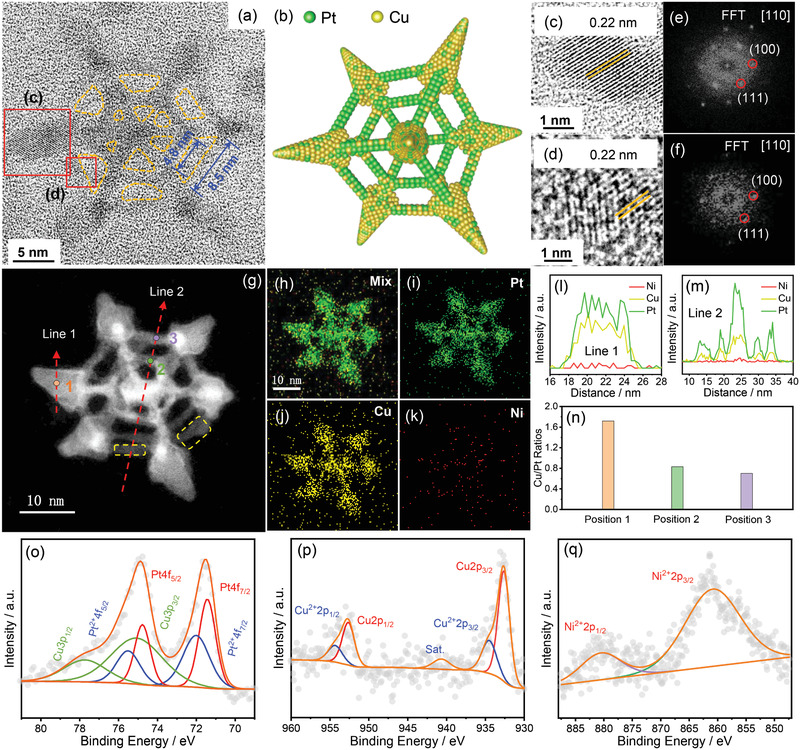
a) HRTEM image of single acid‐treated PtCu NSC. Considering orientations, real edge‐length (*R*) can be expressed as: *R* = *L* $\times \;\sqrt{2}$, where *L* is measured edge‐length. b) Corresponding model schematic illustration. c,d) Enlarged HRTEM images of the selected areas shown in (a). e,f) Corresponding FFT patterns. g) STEM image of single acid‐treated PtCu NSC. h–k) Corresponding EDS elemental mappings. l,m) EDS line scan profiles along red dotted arrow directions in (g). n) EDS elemental ratios of specific positions in (g). o) Elemental Pt, p) Cu, and q) Ni XPS spectra for PtCu A‐NSCs/C.

Nested skeleton cube nanocrystals were also analyzed in the same way: HRTEM image of an acid‐treated skeleton structure reveals a smaller skeleton cube nested entirely within a larger skeleton cube (**Figure** **3**a). The edge‐lengths of the inner and outer skeleton cubes are estimated to be 6.8 and 12.0 nm, respectively. Edge thickness in both inner and outer skeleton cubes is measured to be roughly 2.0 nm (Figure S7, Supporting Information). A spike protrudes outward at each vertex of the outer skeleton cube. The lighter regions bounded by darker edges in the HRTEM image of the hexagonal structure (highlighted in orange in Figure 3a) are confirmed to be hollow channels in the nanocrystal. Amplified HRTEM images of the spike and the ridge of the skeleton cube (marked with red‐squares in Figure 3a) indicate continuous lattice fringes (Figure 3c,d), suggesting high crystallinity. The interplanar spacing of 0.22nm is congruent with that of {111} facets of fcc PtCu alloys, which can be seen in the FFT patterns along [110] directions (Figure 3e,f). Observed XRD diffraction peaks of acid‐treated PtCu NSCs/C (PtCu A‐NSCs/C, see Figures S3b and S4, Supporting Information) are also similar to those of fcc Pt_50_Cu_50_ alloys (PDF#48‐1549). This suggests that although both OSs and NSCs resemble fcc PtCu alloys, the {111} and {100} facets are present in both the spikes and the ridges of the PtCu NSCs in contrast to the {220} facets seen in the PtCu OSs.

The overall elemental compositions of NSCs were measured using EDS and were similar between acid‐treated PtCu NSCs/C and untreated PtCu NSCs/C. In particular, despite the high initial quantity of Ni in the reaction mixture, as‐formed NSCs have very low Ni contents, with PtCu A‐NSCs/C having values of 51.5% Cu, 47.6% Pt, and 0.9% Ni, while PtCu NSCs/C has values of 54.8% Cu, 44.3% Pt, and 1.2% Ni. STEM‐EDS elemental mappings of NSCs (Figure 3h–k) show the mostly well‐mixed distribution of Pt (green), Cu (yellow), and Ni (red) throughout the whole nanocrystal (Figure 3g). EDS line scan profiles (Figure 3l,m) were taken across spike and ridge regions (highlighted in red in Figure 3g). Across spike regions, local composition is shown to be enriched for Cu with a Cu/Pt ratio of 1.72 (position 1 Figure 3g,n), although the distribution of Cu and Pt seems even across the spike (Figure 3l). However, along the ridges, the alloy is enriched for Pt, with Cu/Pt ratios of 0.83 (position 2 in Figure 3g,n) and 0.70 (position 3 in Figure 3g,n). Moreover, EDS line scan across ridge regions (Figure 3m) shows an effective Pt‐rich structure on the surface of the ridges. The Pt4f and Cu2p XPS spectra in the sample before and after the acid treatment show the presence of both the metallic and oxidized states of Pt and Cu (Figure 3o,p; Figure S8a,b, Supporting Information),^[^ ^20^ ^]^ although the metallic form is most common, confirming their presence as an PtCu alloy. On the other hand, Ni can be seen from the Ni2p XPS spectra to be present in Ni^2+^ form (Figure 3q; Figure S8c, Supporting Information),^[^ ^21^, ^22^ ^]^ suggesting that Ni is not incorporated into the alloy.

### 2.2 Ni^2+^‐Directed Anisotropic Growth of PtCu NSCs

**Figure 4:**
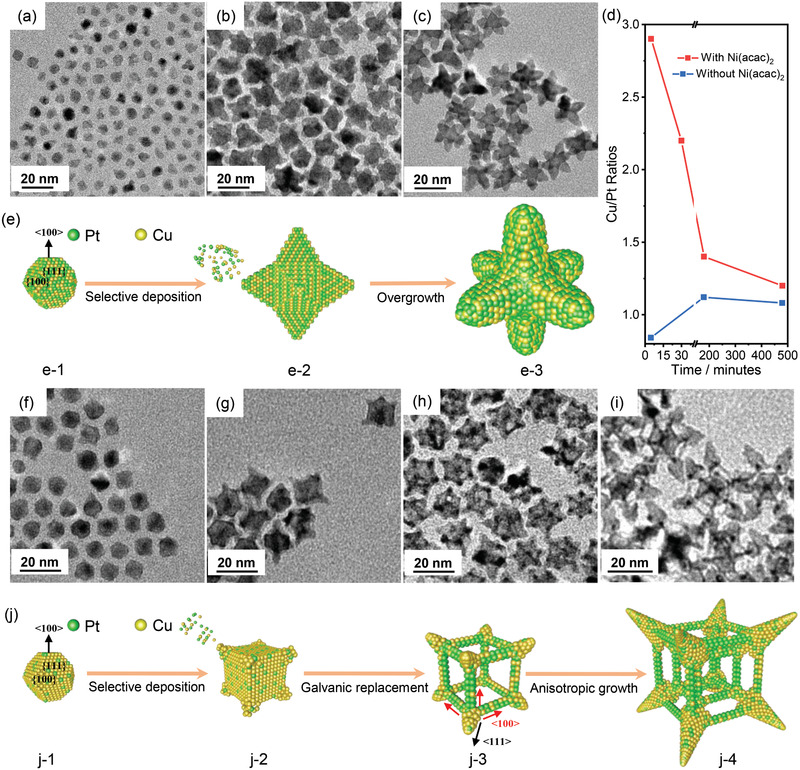
TEM images of time‐dependent intermediates for a–c) solid PtCu OSs and f–i) PtCu NSCs: a,f) 5 min, g) 30 min, b,h) 3 h, and c,i) 8 h. d) The variation of the composition in intermediate products with reaction time. The 3D schematic illustration for the growth of nanocrystals including e) solid PtCu OSs and j) PtCu NSCs.

To understand the role of Ni in the generation of drastically different nanocrystalline structures above, we tracked the growth trajectories of PtCu OSs/NSCs by characterizing the products at intermediate timepoints under controlled reaction conditions using TEM and EDS. **Figure** **4**a–c shows representative TEM images of several intermediates for solid OSs. By 5 min, 6.2 nm spherical nanoparticles had formed with a Cu/Pt ratio of 0.84 (Figure 4a,d). After 3 h, the nanocrystals began to take on an octahedral star shape (Figure 4b), with six symmetric points appearing. The nanocrystals were enriched for Cu, with a Cu/Pt ratio of 1.10 (Figure 4d). After 8 h, the product was close to the 3 h intermediate in shape (Figure 4c) and composition (Figure 4d) though the arm length of the star shape increased from 7.6 nm at 3 h to 10.3 nm at 8 h.

Four intermediate stages were identified for NSCs from TEM imaging (Figure 4f–i). After 5 min, 7.1 nm near‐spherical nanoparticles were observed (Figure 4f) with a Cu/Pt ratio of 2.9 (Figure 4d). By 30 min, 18.9 nm (edge‐to‐edge size) nanocubes were observed with spikes protruding from the vertices (Figure 4g), though the cubes were still solid. The Pt content in the nanocrystals was increased as compared with the 5 min intermediates, with a Cu/Pt ratio of 2.2 (Figure 4d). By 3 h, the centers of the nanocrystals have started to hollow out and form a skeleton structure (Figure 4h) and the Pt composition has increased, with a Cu/Pt ratio of 1.4 (Figure 4d). Meanwhile, their size was relatively unchanged. From 3 to 8 h, the skeleton cube intermediates grew anisotropically into nested skeleton structures, forming outer skeleton cubes with an edge‐to‐edge size of 28.3 nm and symmetric spikes of edge‐length of 11.3 nm at vertices (Figure 4i). The Pt composition was only slightly increased, with the final Cu/Pt ratio of 1.2 (Figure 4d). Throughout, by monitoring the relative composition of Cu/Pt/Ni in these intermediate products (Figure S9, Supporting Information), we found very low Ni composition (roughly 1.2%) throughout the reaction. XPS analysis showed only the presence of Ni^2+^ signal in PtCu NSCs.^[^ ^21^, ^22^ ^]^ It suggests that Ni^2+^ species are absorbed on the surface of the intermediate products and play catalytic role in reaction solution in the growth of these NSC nanocrystals.

### 2.3 Growth Mechanisms of PtCu OSs/NSCs

Based on observations of the intermediates at different timepoints and the detailed analysis of the structure of the as‐prepared product, we proposed a mechanism of the formation of these two different nanocrystals (Figure 4e,j). Without the presence of Ni precursor, the higher standard reduction potential of Pt^2+^/Pt (1.18 V) compared to Cu^2+^/Cu (0.34 V) led to a faster reduction rate for Pt, forming Pt_54.3_Cu_45.7_ alloys which are enriched for Pt. Such thermodynamic arguments have been proposed for the formation of other stable alloy phases, with H. Peng et al. showing that about 7 nm PtCu nanocrystals have a stable composition of 58% Pt and 42% Cu.^[^ ^23^ ^]^ Initially, energy minimization caused these nanocrystals to form as Wulff polyhedron structures (Figure 4e‐1).^[^ ^24^, ^25^ ^]^ On the other hand, Br¯ have been shown to guide the codeposition of Pt and Cu for the formation of hexapod nanostructures.^[^ ^26^ ^]^ Therefore, we suppose that Br¯ derived from the CTAB surfactant can adsorb preferentially on {111} facets of Pt‐rich Wulff seeds and drive the increased codeposition of Pt and Cu along <100> directions. Moreover, the intermediates formed from this preferential deposition retained octahedral symmetry, passing thought a putative Wulff intermediate to the observed intermediate at 3 h with clear octahedral symmetry (Figure 4e‐2). Continued overgrowth along the <100> directions led to the extension of the protrusions on these facets into the arms of the octahedral star seen in PtCu OSs (Figure 4e‐3).

By comparison, in the presence of Ni precursor, an alternative mechanism was proposed (Figure 4j). Ni^2+^ acts as a catalyst to increase the reduction rate of Cu^2+^. Despite the higher standard reduction potential of Pt^2+^/Pt (1.18 V) than that of Cu^2+^/Cu (0.34 V), kinetic selectivity led to reduction of Cu^2+^ dominating in the initial nucleation step. Therefore, nanocrystals formed were Cu‐rich Wulff polyhedrons (Figure 4j‐1). The high concentration of Cu would lead to Br¯ adsorbed on {100} facets of Cu‐rich Wulff seeds, followed by the formation of cubic intermediates.^[^ ^20^, ^27^ ^]^ These nanocrystals then exhibited preferential growth directions to form cubic intermediates with protrusions at the vertices by a combination of kinetic and thermodynamic effects (Figure 4j‐2). This would account for the appearance of the Cu‐rich nanocubes with the protrusions at the vertices after 30 min of reaction time. Sequentially, the higher standard reduction potential of Pt^2+^/Pt caused galvanic replacement of Cu by Pt^2+^. Once again, the reaction was fastest on the Cu‐rich {100} facets. Surfactant molecules (OAm and CTAB) have been shown to guide the formation of Pt‐rich nanorods,^[^ ^28^, ^29^ ^]^ in our case, which can direct the deposition the resulting Pt atoms on the edges of cubes (along <100> directions) to form Pt‐rich rods (position 2 in Figure 3g,n). This resulted in the formation of a hollowed‐out skeleton cube, as seen in the 3 h intermediate (Figure 4j‐3). In particular, the two‐phase reaction of Cu‐deposition followed by Pt galvanic replacement can account for the Pt‐enriched surface of the ridges of the skeleton cube. As the reaction proceeded, Cu‐deposition taken place mainly on the spikes on the vertices, on account of the kinetics‐driven preferential galvanic replacement by Pt on {100} facets, leading to the continued growth of the vertex spikes with a relatively enriched Cu content, which was consistent with our observation that the vertex spikes of the final product are enriched for Cu (position 1 in Figure 3g,n). In addition, a second outer layer of Pt‐enriched ridges were formed between the Cu‐rich vertex spikes. These ridges are even higher in Pt contents than the ridges formed in the 3 h intermediate (positions 2 and 3 in Figure 3g,n). We suppose that these surfactant molecules (OAm and CTAB) can provide rod‐like templates for the deposition of Pt and Cu on Cu‐rich surface along <100> directions, eventually directing the growth of Pt‐rich rods spanning the spikes for forming an external skeleton cube (Figure 4j‐4). Furthermore, additional ridges can be seen forming in some NSC nanocrystals (highlighted in yellow, Figure 3g), which is both suggestive of that the ridges of the external skeleton cubes are individually formed and potentially indicates that with further optimization of our synthetic approach, a higher‐order multilayered nesting structure might be achievable.

### 2.4 Electrocatalytic ORR Performances

Motivated by observations of greatly enhanced catalytic activity for ORR in single‐layered skeleton structures,^[^ ^9^, ^27^ ^]^ we wondered if the PtCu NSCs we prepared can deliver even better performances. To examine this, we evaluated the ORR performances of our as‐prepared PtCu NSCs/C, PtCu OSs/C, and their acid treated forms (PtCu A‐NSCs and PtCu A‐OSs/C) in 0.1 m HClO_4_ solution. We also evaluated the ECSAs of these catalysts using CO‐stripping experiments (Figure S9, Supporting Information). Further, we compared these values mentioned above with commercially available Pt/C as a benchmark.

**Figure 5:**
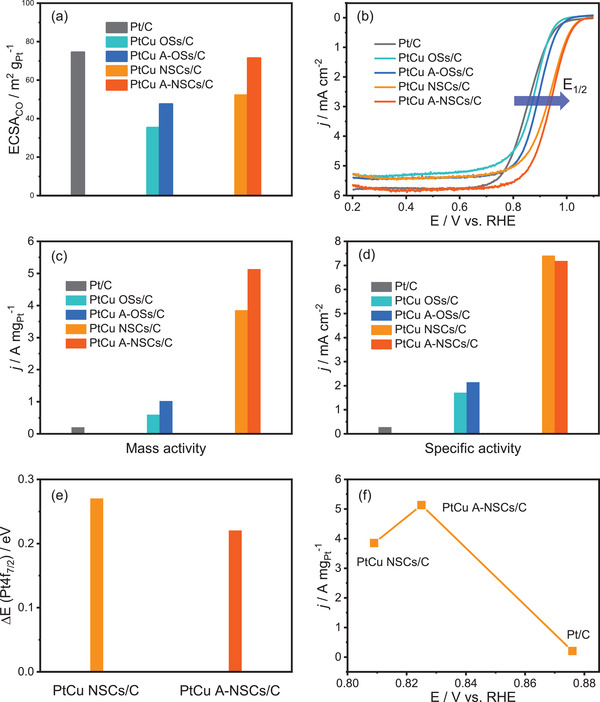
a) ECSAs and b) the polarization curves of PtCu OSs/C, PtCu NSCs/C before and after the acetic acid treatment, and Pt/C as a comparison. ECSAs were calculated by the integration of CO‐stripping charges. The polarization curves were collected in O_2_‐saturated 0.1 m HClO_4_ solution at a rotation rate of 1600 rpm and at a sweep rate of 10 mV s^–1^. At 0.9 V versus RHE, c) mass activity, and d) specific activity for these five catalysts. e) The relative binding energy (Δ*E* (Pt4f_7/2_)) of Pt4f_7/2_ XPS states can be expressed as: Δ*E* (Pt4f_7/2_) = *E* (Pt4f_7/2_) − 71.2 eV, where *E* (Pt4f_7/2_) is the binding energy of Pt4f_7/2_ XPS states for PtCu NSC catalysts; the binding energy of 71.2 eV is attributed to the Pt4f_7/2_ state for pure Pt.^[^ ^36^ ^]^ f) The correlation between the mass activity and the CO‐stripping potential.

It can be seen that alloy catalysts display higher ECSA_CO_ after the acid treatment due to the cleaning of the surface (**Figure** **5**a; Figure S10, Supporting Information). The ECSA_CO_ of PtCu A‐NSCs/C (71.4 m^2^ g_Pt_^–1^) is much higher than PtCu NSCs/C (52.2 m^2^ g_Pt_^–1^). It is also higher than PtCu A‐OSs/C (47.6 m^2^ g_Pt_^–1^), which in turn has a higher ECSA_CO_ than PtCu OSs/C (35.3 m^2^ g_Pt_^–1^). Similarly, we also measured the ECSA_Hupd_ (Figure S11, Supporting Information), which has the consistent trend of change with the ECSA_CO_ of catalysts.

Plotting polarization curves of the ORR for all five catalysts, we found that four PtCu samples have a more positive half‐wave potential (*E*_1/2_) compared to the Pt/C catalyst (Figure 5b). At 0.9 V versus RHE, PtCu A‐NSCs/C catalyst (Figure 5c) shows the greatest mass activity (5.13 A mg_Pt_^–1^), 25.7 times higher than Pt/C (0.2 A mg_Pt_^–1^). PtCu A‐NSCs/C also has 1.3 times greater mass activity than nonacid‐treated PtCu NSCs/C. On the other hand, both acid‐treated and non‐acid‐treated OSs have a substantially lower than PtCu A‐NSCs/C by 5.0 (1.02 A mg_Pt_^–1^) and 8.7 (0.59 A mg_Pt_^–1^) times, respectively. Importantly, PtCu NSC catalysts show much higher specific activity than solid PtCu OS catalysts and Pt/C (Figure 5d). The specific activity of PtCu NSCs/C catalysts are similar before and after the acid treatment (7.4 vs 7.2 mA cm^–2^), about 27 times higher than Pt/C (0.27 mA cm^–2^). Furthermore, we compared the ORR activity of the nested skeleton structures we prepare and the single‐layered Pt‐alloyed skeletons reported recently. Despite their superficially similar hollow structures, our PtCu skeleton cubes with the nested structure show higher mass activity and specific activity (see Table S1 in the Supporting Information).

The enhanced activity of PtCu A‐NSCs/C catalyst with respect to the untreated PtCu NSCs/C and commercial Pt/C catalysts can be rationalized by the d‐band center theory tuning the adsorption strength of various oxygenated intermediates. Combined the early reports,^[^ ^30^, ^31^, ^32^ ^]^ Pt‐rich surface in our PtCu A‐NSCs/C catalyst shows 2.87% of compressive strain, which is mainly responsible for the optimization of the downshifted Pt d‐band center in energy, weakening the adsorption of oxygenated intermediates on Pt sites. The downshifted Pt d‐band center has been shown to correspond to increased binding energy of Pt4f XPS states.^[^ ^33^, ^34^, ^35^ ^]^ Our XPS analyses (Figure 5e) showed increased Pt4f_7/2_ binding energies of up to 0.27 and 0.22 eV for PtCu NSCs/C and PtCu A‐NSCs/C compared to pure Pt,^[^ ^36^ ^]^ respectively. Therefore, we can deduce that the position of Pt d‐band center for PtCu A‐NSCs/C is intermediate between PtCu NSCs/C and Pt/C. This trend in d‐band center was also confirmed by the CO‐stripping experiments. A downward shift in the Pt d‐band center led to weakening of Pt‐CO interaction and manifested as a lower CO‐stripping potential.^[^ ^37^, ^38^ ^]^ In our CO‐stripping experiments, PtCu A‐NSCs/C (0.825 V) has an intermediate potential between PtCu NSCs/C (0.809 V) and Pt/C (0.876 V), confirming PtCu A‐NSCs has an intermediate Pt–CO binding energy between PtCu NSCs/C and Pt/C (Figure 5f). Electrochemical ORR surveys, XPS and CO‐stripping experiments are all consistent with the Pt d‐band center of PtCu A‐NSCs/C located between those of PtCu NSCs/C and Pt/C. We can interpret these results through the lens of the Sabatier principle:^[^ ^39^ ^]^ PtCu A‐NSCs/C possesses the optimum binding energy to the oxygenated intermediates relative to PtCu NSCs/C and Pt/C, and consequently shows the highest mass ORR activity (Figure 5f). It is worth noting that a similar effect is observed to a lesser extent for PtCu OSs, where PtCu A‐OSs/C has the optimal binding energy for oxygenated intermediates, lying between PtCu OSs/C and Pt/C, and thus giving rise to the substantial enhancement in mass activity (Figure S12, Supporting Information).

Furthermore, we also considered the origin of the enhancement of the catalytic activity of PtCu NSCs compared to PtCu OSs. Our EDS and XPS results showed a very low amount of Ni^2+^ species in our NSCs, and thus suggesting that the activity enhancement is unrelated to the use of Ni precursor in the synthetic process. Instead, we believe that the enhancement is on account of the presence of nested skeleton structures. The hollow cavities in NSCs allow for substantial mass‐transfer in ORR catalysis. Besides, ultrafine 1D Pt‐enriched nanowires which make up of the ridges of the nested cubes present much more catalytically active Pt than the well‐mixed PtCu alloy arms of the octahedral star.

**Figure 6:**
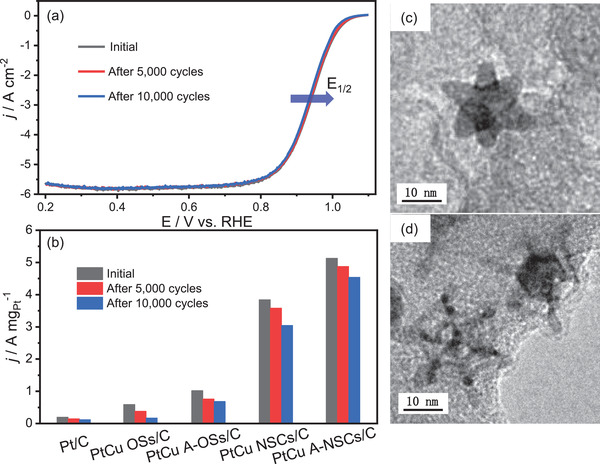
a) The polarization curves of PtCu A‐NSCs/C after 5000/10 000 cycles. b) At 0.9 V versus RHE, the comparison of mass activity for all five catalysts after 5000/10 000 cycles. TEM images of c) PtCu A‐OSs/C and d) PtCu A‐NSCs/C after 10 000 cycles.

Finally, we evaluated the ORR stability of various catalysts in O_2_‐saturated 0.1 m HClO_4_ solution at a scan rate of 100 mV s^–1^. By comparison of the E_1/2_ (**Figure** **6**a; Figure S13, Supporting Information) and mass activities (Figure 6b) of various catalysts, we note that PtCu A‐NSCs/C catalyst showed greatly improved ORR stability after 5000/10000 cycles. After 10 000 cycles, the decays in E_1/2_ and mass activity for PtCu A‐NSCs/C reached ≈6 mV and 11.5%, respectively, while the decays for the other three alloy catalysts were considerably greater (Table S2, Supporting Information). We propose that this greater stability can be attributed to the synergistic effects of the shape of our nanoalloys and the acid treatment process. TEM images (Figure 6c,d; Figure S14, Supporting Information) showed the OSs and NSCs retained their shape well after 10 000 cycles. This can be rationalized by proposing that the protruding arms in OSs and the vertex spikes in NSCs might suppress the 3D structural collapse and agglomeration of the nanoparticles, leading to increased stability for NSCs, and to a lesser extent OSs. Moreover, the compressive Pt‐rich surface in PtCu nested structures can provide a barrier for bulk Cu diffusion to suppress dealloying under the conditions the ORR stability tested, resulting in the high stability toward the ORR. This could be seen from our EDS results, where the Cu content decreased by 4.5% for PtCu A‐NSCs/C, and 7.1% for PtCu A‐OSs/C, while the loss was greater for PtCu NSCs/C (9.5%) and PtCu OSs/C (12.3%) after 10000 cycles.

## 3 Conclusions

We have developed a one‐pot wet chemical method which can be switched between the production of PtCu nanocrystals of two drastically different 3D structures (solid OSs and NSCs) by the simple addition of Ni(acac)_2_. In the absence of Ni(acac)_2_, solid PtCu OSs were obtained but when Ni(acac)_2_ was added the product switched to PtCu NSCs. Our studies on growth mechanism suggested that Ni^2+^ acted as a catalyst, accelerating the reduction of the Cu precursor, and acted as a structure‐directing agent as well, boosting the anisotropic growth of PtCu skeleton cube structures by acting in concert with the thermodynamic effects of surface energy minimization. This anisotropic growth mechanism allowed the formation of nested layers of PtCu skeletons, a novel structure in PtCu alloy nanocrystals. This novel nested structure greatly enhanced ORR activity, far higher than that of solid PtCu OS catalysts and commercially available Pt/C. We revealed that enhanced ORR activity varied depending on the 3D structure of alloy catalysts. The acetic acid treatment could expose more active sites, and thus optimizing the binding strength of the oxygenated species. Moreover, we proposed that the surface protrusions and vertex spikes formed in our nanocrystals can counteract the structural collapse and the agglomeration of catalysts under ORR stability tests and be observed to maintain their 3D structures postexperiment. Finally, our observations suggest that even higher‐order multilayered nesting structures are possible with further optimization of synthesis protocols and this work may offer a starting point for the rational design and synthesis of multilayered alloy skeletons for other electrocatalytic applications.

## 4 Experimental Section

### Materials

Platinum acetylacetonate (Pt(acac)_2_, Pt 48 wt%), copper acetylacetonate (Cu(acac)_2_, 97%), nickel acetylacetonate (Ni(acac)_2_, 95%), cetyltrimethyl ammonium bromide (CTAB, 99%), and Nafion (5 wt%) were purchased from Alfa Aesar. Oleylamine (OAm, 80–90%) was purchased from Aladdin. Hexane, ethanol, and Perchloric acid (HClO_4_, 70–72%) were purchased from Sinopharm Chemical Reagent Co. Ltd. China. These materials purchased were not purified in this experiments. Ultrapure water (18.2 MΩ cm^–1^) was used in this experiments.

### Synthesis of PtCu NSCs

17 mg Pt(acac)_2_, 34 mg Cu(acac)_2_, 8–14 mg Ni(acac)_2_, and 100 mg CTAB were added to 7 mL OAm solution in a two‐neck flask and heated to 175 ℃ for 8 h under magnetic stirring in an oil bath after ultrasonic treatment. After the end of the reaction, the product was allowed to cool to room temperature and a mixed solution of hexane and ethanol was used to wash the product obtained. This process was repeated five times with the product being separated by centrifugation. Finally, the black sample was re‐dispersed in hexane.

### Synthesis of Solid PtCu OSs

The same method was used to prepare solid PtCu OSs, except without the introduction of Ni(acac)_2_ to OAm solution.

### Acetic Acid Treatment for Catalysts

PtCu OSs/NSCs were first supported on carbon black (PtCu OSs/C and PtCu NSCs/C), followed by dispersion in an acetic acid solution. This mix solution was heated to 70 ℃ for 10 h under magnetic stirring to obtain final catalysts (PtCu A‐OSs/C and PtCu A‐NSCs/C).

### Material Characterizations

A FEI Tecnai F20 instrument and a Titan Themis G3 ETEM instrument equipped with a spherical‐aberration corrector were used to collect the transmission electron microscope (TEM) images of samples loaded on Mo grids. The spherical‐aberration corrected scanning transmission electron microscope (STEM) images and the energy dispersive X‐ray spectroscopy (EDS) mappings/line‐scans of samples were obtained on a JEM ARM 200F instrument at 200 kV. The XRD instrument with a Cu‐K*α* source was used to characterize the crystal pattern of samples. The elemental XPS spectrum of samples was collected on a Thermo Scientific K‐Alpha instrument equipped with an Al K*α* source. The ICP‐OES (Agilent 5110) instrument was used to quantify elemental composition in samples.

### Electrochemical Characterizations

All catalysts were first loaded on glassy carbon dish electrodes with a diameter of 5 mm (Pt loading content: 2.6 µg cm^–2^ for PtCu alloy nanoskeleton catalysts, 6.8 µg cm^–2^ for solid PtCu alloy catalysts, and 10.2 µg cm^–2^ for commercial Pt/C catalyst). Saturated Ag/AgCl and Pt wire electrodes were acted as the reference and counter electrodes, respectively. Cyclic voltammetry (CV) curves were measured in N_2_‐purged 0.1 m HClO_4_ solution at a scan rate of 100 mV s^–1^. The polarization curves were measured in O_2_‐purged 0.1 m HClO_4_ solution at a scan rate of 10 mV s^–1^ at 1600 rmp. The kinetic current was calculated at 0.9 V versus RHE (reversible hydrogen electrode) according to K–L equation.^[^ ^40^ ^]^ The ORR stability tests were conducted in O_2_‐purged 0.1 m HClO_4_ solution at a scan rate of 100 mV s^–1^. The CO‐stripping CV curves were measured in N_2_‐purged 0.1 m HClO_4_ solution at a scan rate of 50 mV s^–1^ after CO molecules adsorbed on catalysts. All electrochemical tests were carried out at room temperature.

## Conflict of Interest

The authors declare no conflict of interest.

## Supporting information

Supporting Information

## Data Availability

Research data are not shared.
