# Supplementary material for: Ni2+‐Directed Anisotropic Growth of PtCu Nested Skeleton Cubes Boosting Electroreduction of Oxygen
Source: Adv Sci (Weinh). 2022 Mar 10;9(14):2104927. doi: 10.1002/advs.202104927 (PMC9108632; doi:10.1002/advs.202104927)
Supplement: Supplementary file 1 — Supporting Information [file ADVS-9-2104927-s001.pdf]

ADVANCED SCIENCE
Open Access

Supporting Information

for *Adv. Sci.*, DOI 10.1002/advs.202104927

Ni^{2+}-Directed Anisotropic Growth of PtCu Nested Skeleton Cubes Boosting Electroreduction of Oxygen

Yafeng Zhang, Kai Ye, Qianru Liu, Juan Qin, Qike Jiang, Bing Yang and Feng Yin**

ADVANCED SCIENCE
Open Access

Supporting Information

for *Adv. Sci.*, DOI: 10.1002/advs.202104927

Ni^{2+}-Directed Anisotropic Growth of PtCu Nested Skeleton Cubes Boosting Electroreduction of Oxygen

Yafeng Zhang, Kai Ye, Qianru Liu, Juan Qin, Qike Jiang, Bing Yang*, Feng Yin*

Supporting Information

Ni^{2+}-Directed Anisotropic Growth of PtCu Nested Skeleton Cubes Boosting Electroreduction of Oxygen

Yafeng Zhang, Kai Ye, Qianru Liu, Juan Qin, Qike Jiang, Bing Yang*, Feng Yin*

Y. Zhang, K. Ye, Q. Liu, J. Qin

School of Physics and Information Technology

Shaanxi Normal University,

Xi’an 710119, China

F. Yin

School of Physics and Information Technology

Key Laboratory of Syngas Conversion of Shaanxi Province

Shaanxi Normal University

Xi’an 710119, China

E-mail: fengyin@snnu.edu.cn

B. Yang

CAS Key Laboratory of Science and Technology on Applied Catalysis

Dalian National Laboratory for Clean Energy

Dalian Institute of Chemical Physics

Dalian, 116023, China

E-mail: byang@dicp.ac.cn

Q. Jiang

Dalian National Laboratory for Clean Energy

Dalian Institute of Chemical Physics

Dalian 116023, China

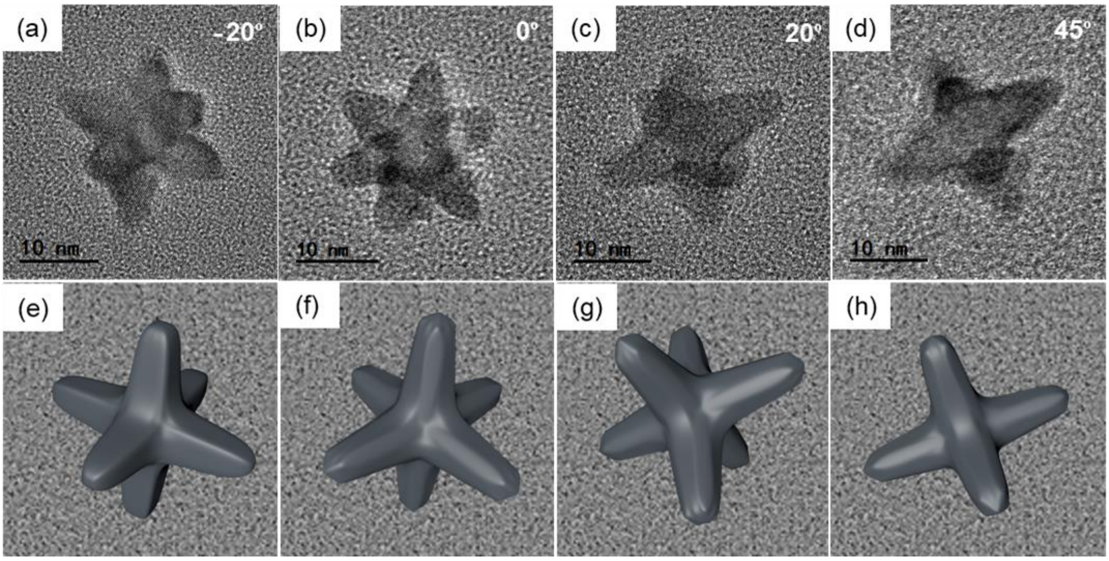

Figure S1. (a-d) TEM images of single PtCu OS at different rotation angles. (e-h) Corresponding 3D model schematic illustration.

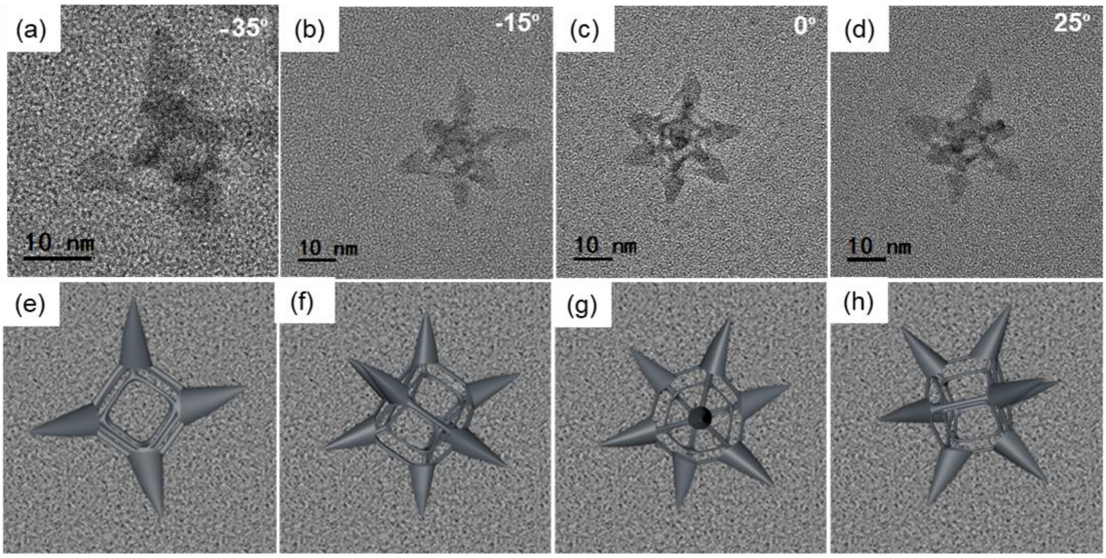

Figure S2. (a-d) TEM images of single PtCu NSC at different rotation angles. (e-h) Corresponding 3D model schematic illustration.

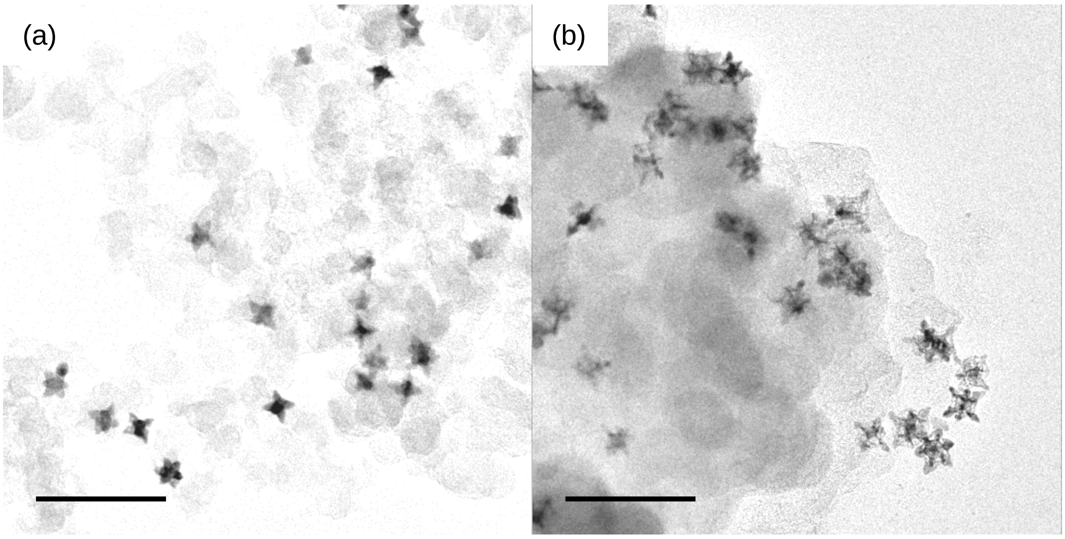

Figure S3. TEM image of (a) PtCu A-OSs/C and (b) PtCu A-NSCs/C catalysts.

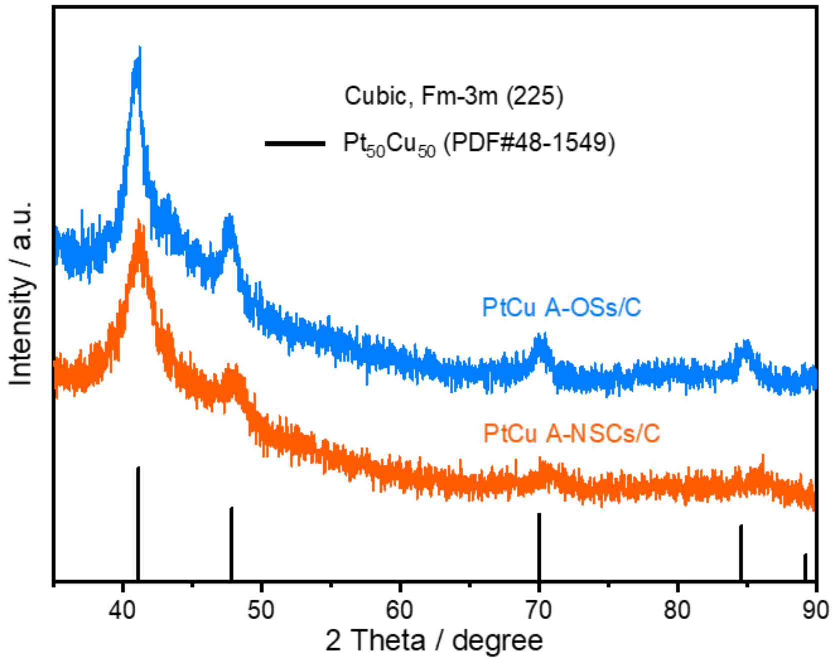

Figure S4. XRD patterns of PtCu A-OSs/C and PtCu A-NSCs/C.

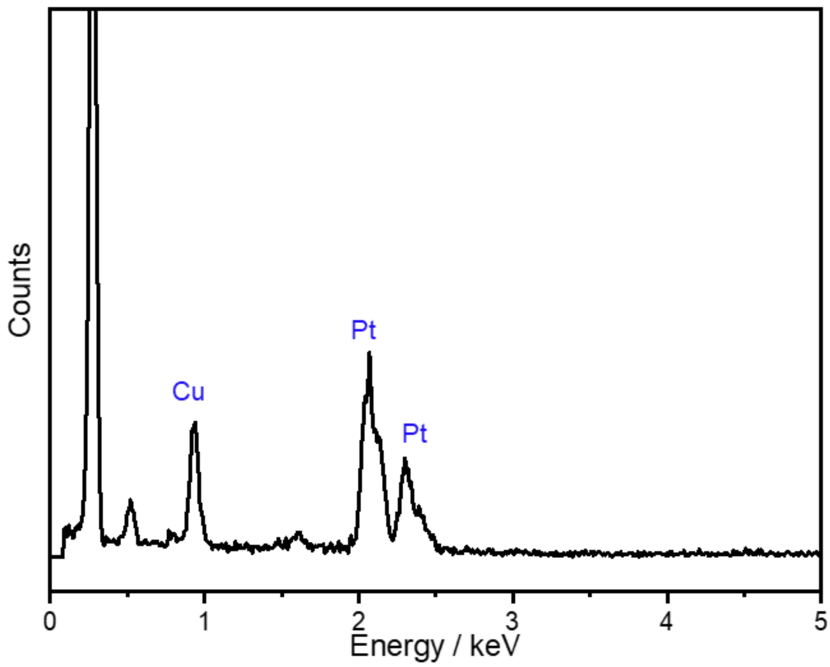

Figure S5. EDS spectrum of PtCu A-OSs/C.

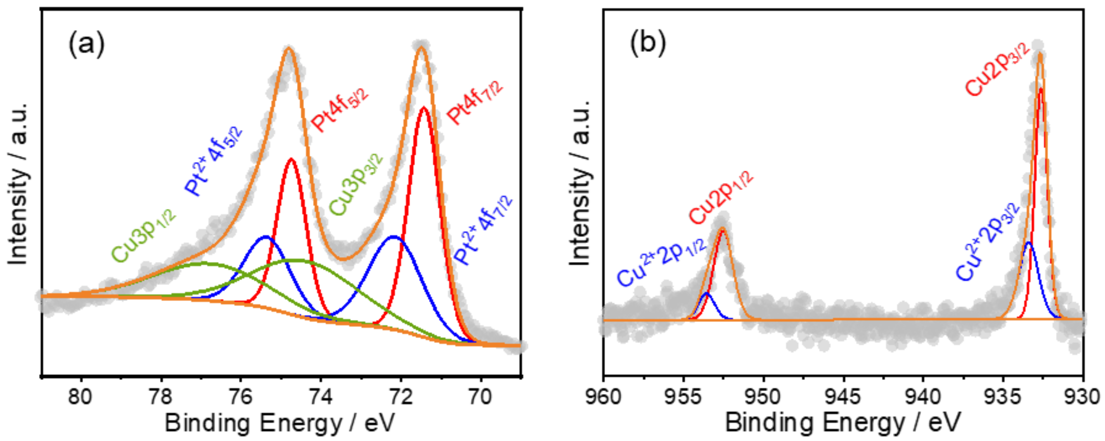

Figure S6. (a) Pt4f and (b) Cu2p XPS spectra for PtCu OSs/C.

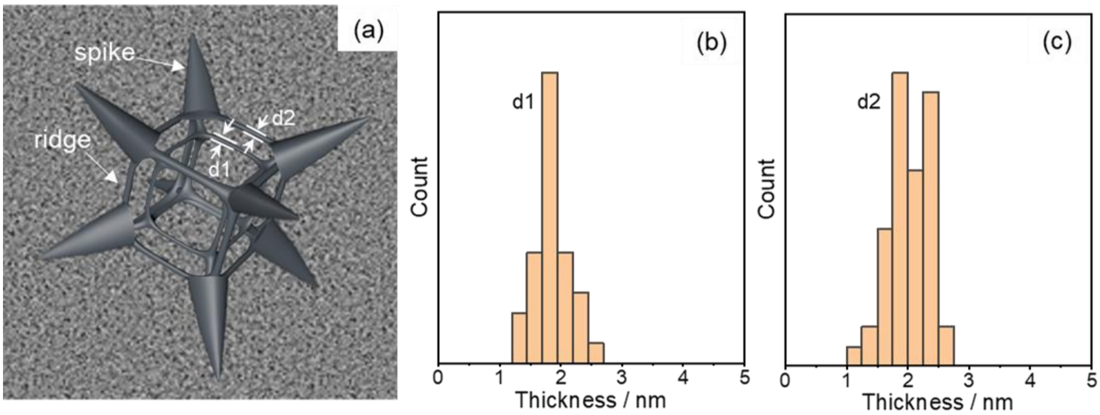

Figure S7. (a) 3D model schematic illustration for PtCu NSCs. (b,c) Size distribution in thickness for ridges.

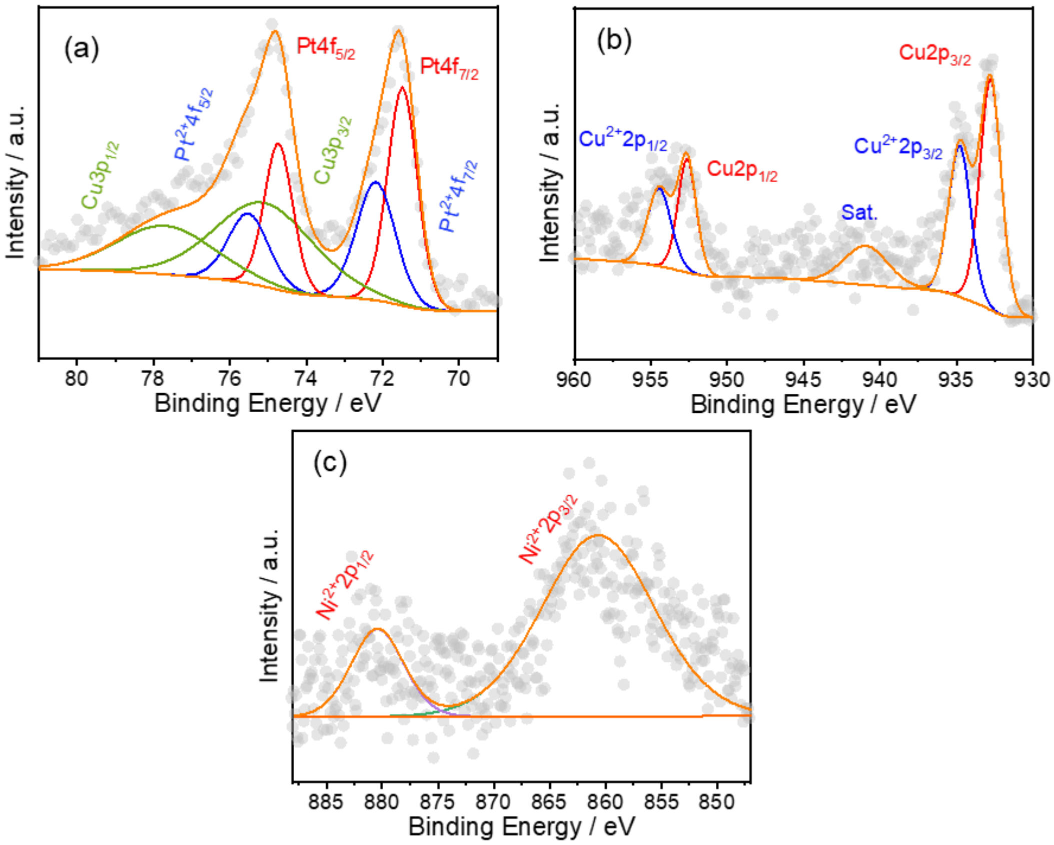

Figure S8. (a) Pt4f, (b) Cu2p, and (c) Ni2p XPS spectra for PtCu NSCs/C.

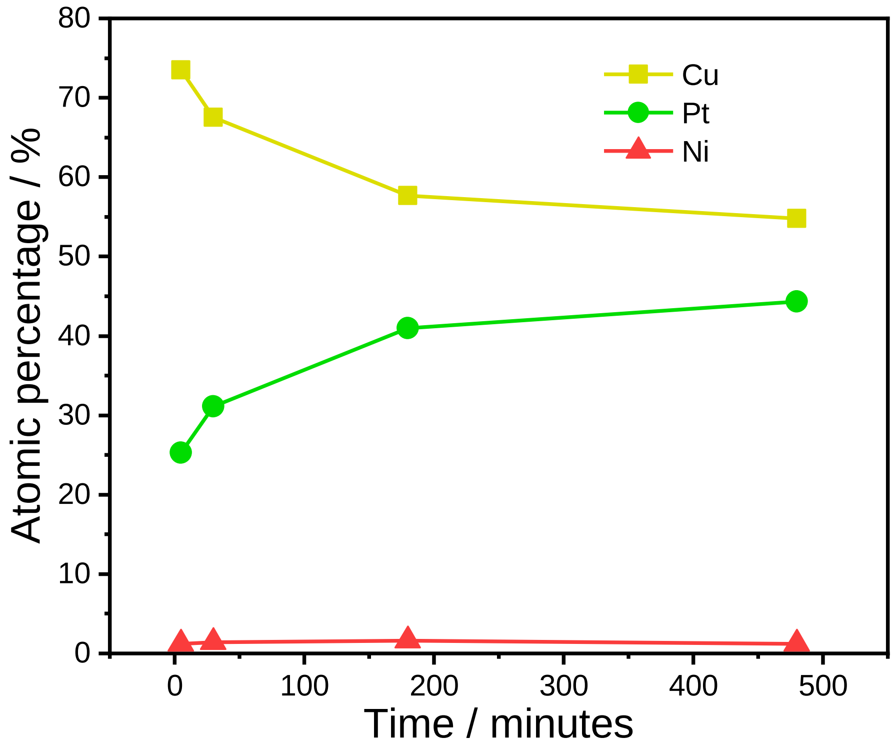

Figure S9. The relation of the composition in intermediates for PtCu NSCs with reaction time.

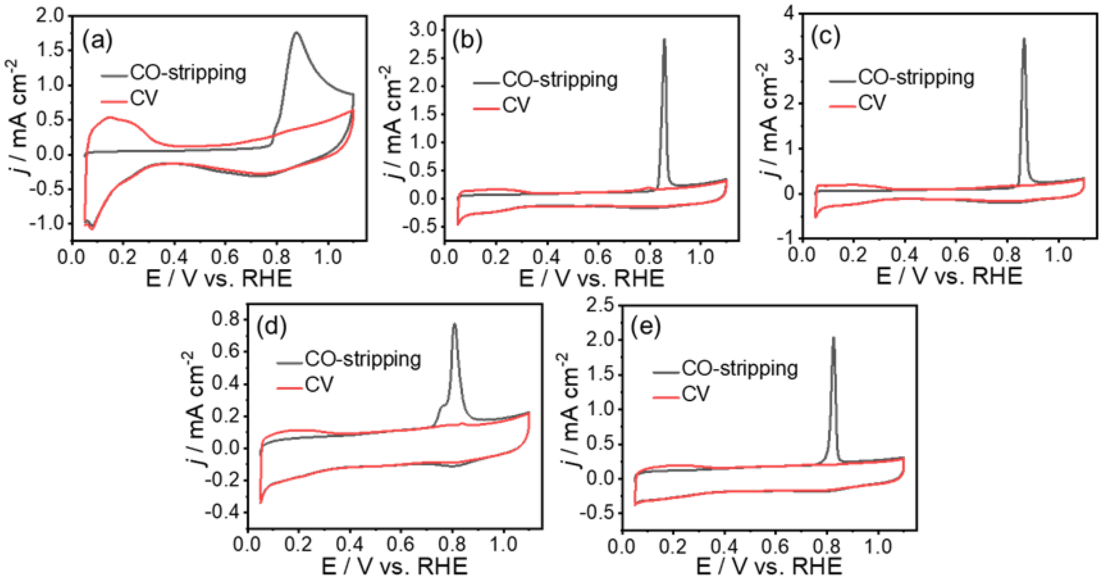

Figure S10. CO-stripping curves of (a) Pt/C, (b) PtCu OSs/C, (c) PtCu A-OSs/C, (d) PtCu NSCs/C, and (e) PtCu A-NSCs/C. CO-stripping curves were collected in N_2-saturated 0.1 M $HClO_4$ solution at a scan rate of 50 mV s^{-1}.

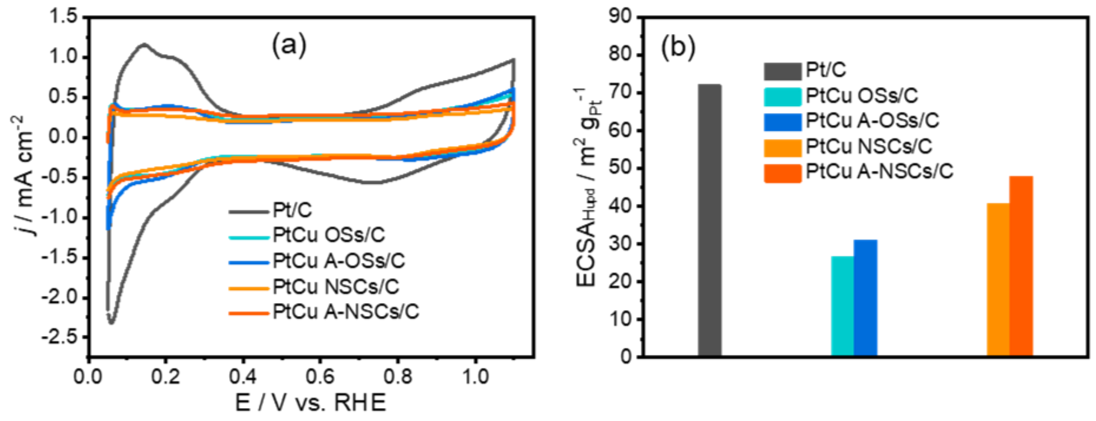

Figure S11. (a) CV curves of Pt/C, PtCu OSs/C, PtCu A-OSs/C, PtCu NSCs/C, and PtCu A-NSCs/C, collected in N_2 saturated 0.1 M $HClO_4$ solution at a scan rate of 100 mV s^{-1}. (b) $ECSA_{Hupd}$ obtained by integrated charge from underpotentially deposited hydrogen in (a).

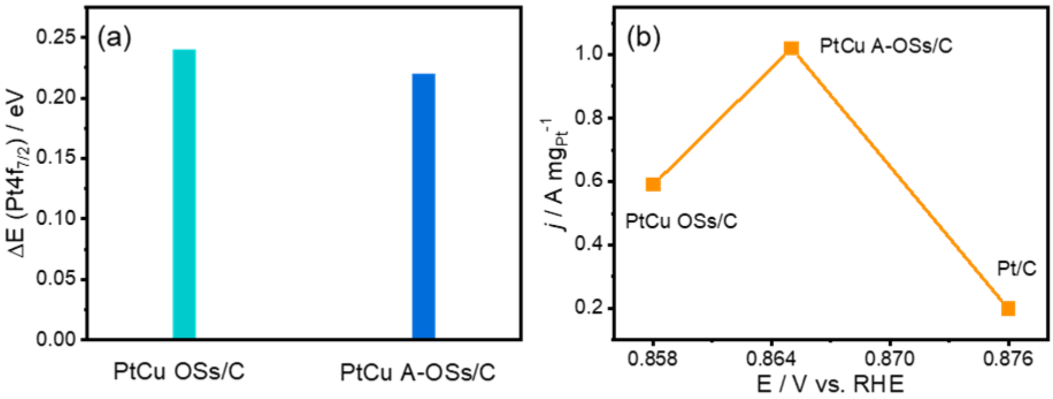

Figure S12. (a) The relative binding energy (ΔE ($Pt4f_{7/2}$)) of $Pt4f_{7/2}$ XPS states can be expressed as: ΔE ($Pt4f_{7/2}$) = E ($Pt4f_{7/2}$) – 71.2 eV, where E ($Pt4f_{7/2}$) is the binding energy of $Pt4f_{7/2}$ XPS states for PtCu OS catalysts; the binding energy of 71.2 eV is attributed to the Pt $4f_{7/2}$ state for pure Pt[1]. (b) The correlation between the mass activity and CO-stripping potential.

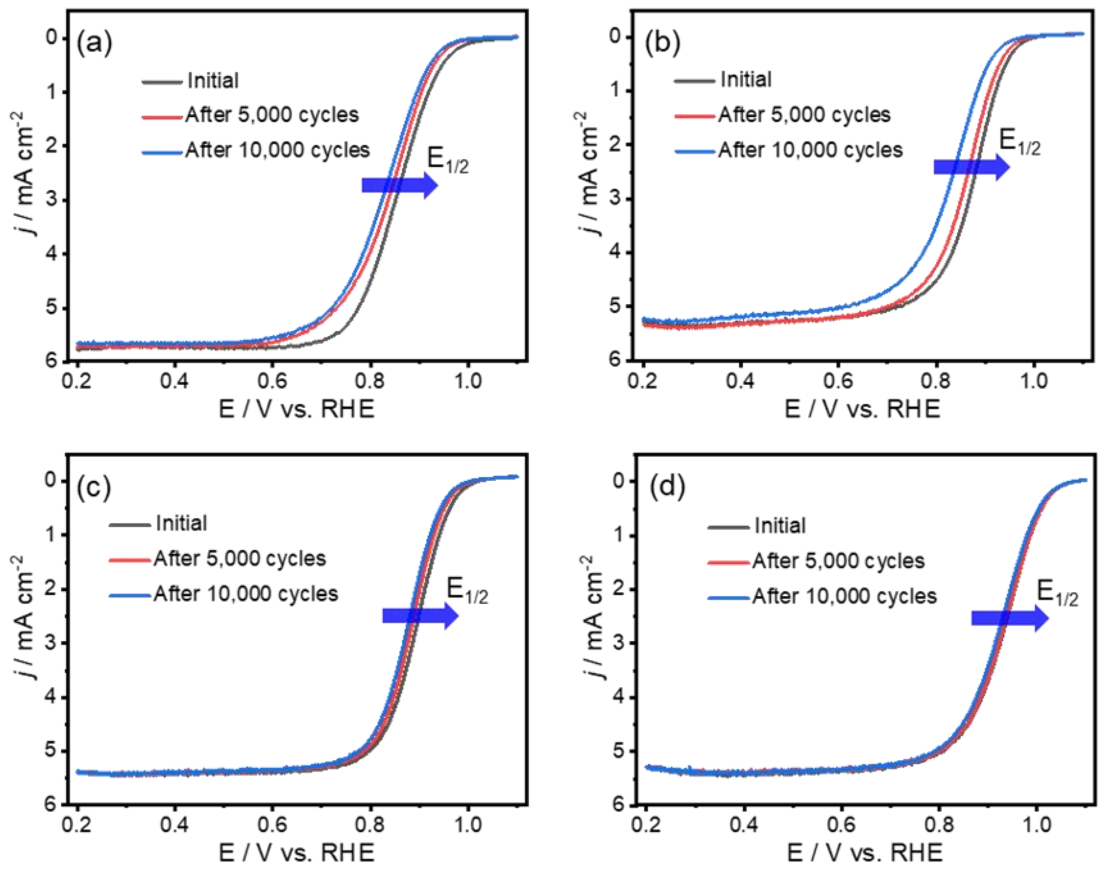

Figure S13. The polarization curves of (a) Pt/C, (b) PtCu OSs/C, (c) PtCu A-OSs/C, and (d) PtCu NSCs/C after 5,000/10,000 cycles.

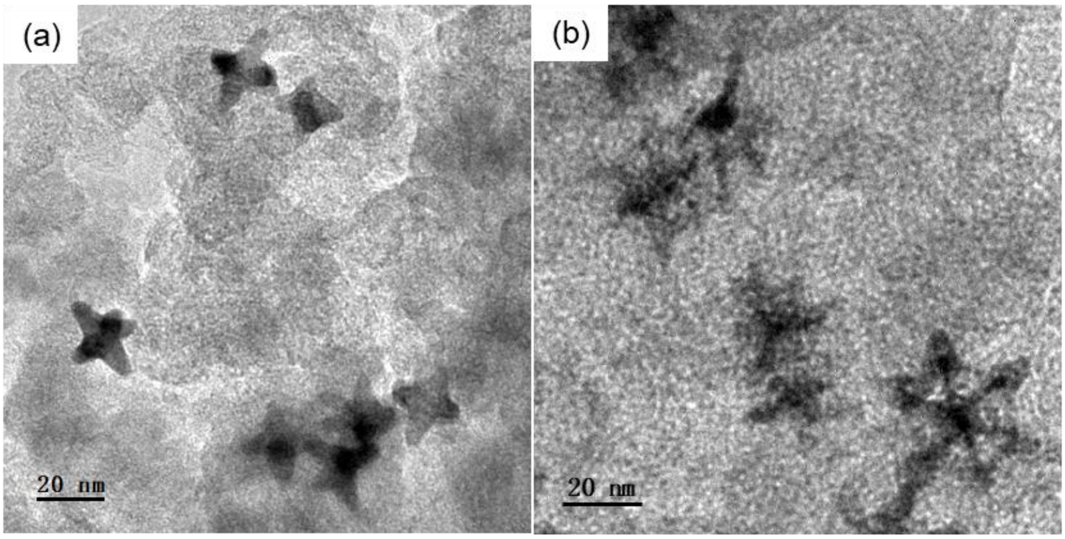

Figure S14 TEM images of (a) PtCu OSs/C and (b) PtCu NSCs/C after 10,000 cycles.

Table S1. Comparison ORR performances of PtCu OS/NSC catalysts in this work with several single-layered Pt-alloyed skeleton catalysts from recent published works. Electrochemical tests were conducted in O_2-purged 0.1 M $HClO_4$ solution. Mass activity and specific activity are calculated at 0.9 V vs. RHE.

Catalysts	Mass activity ($A\ mg_{Pt}^{-1}$)	Specific activity ($mA\ cm^{-2}$)	Ref.
PtCu A-NSCs/C	5.13	7.2	This work
PtCu NSCs/C	3.86	7.4	
PtCu A-OSs/C	1.02	2.14	
PtCu OSs/C	0.59	1.7	
Pt_3Ni rhombic dodecahedral nanoskeletons/C	5.7	8.6	[2]
PtCu octopod nanoskeletons/C	3.26	5.98	[3]
PtNi tetrahexahedral nanoskeletons/C	2.95	6.37	[4]
Ordered PtCu nanoskeletons/C	2.47	4.69	[5]
PdPt tesseracts/C	1.86	2.09	[6]
PtCuCo rhombic dodecahedral nanoskeletons/C	1.56	2.69	[7]
PtCuPd cubic nanoskeletons/C	1.04	2.41	[8]
PtCuNi rhombic dodecahedral nanoskeletons/C	0.86	1.65	[9]
PtCu dodecahedral nanoskeletons/C	0.79	2.03	[10]
Pt cubic nanoskeletons/C	0.45	0.53	[11]

Table S2. Comparison stability of the ORR for various catalysts.

Catalysts	After 10,000 cycles	
	Half-wave potential decay (mV)	Mass activity decay (%)
PtCu A-NSCs/C	6	11.5
PtCu NSCs/C	9	20.8
PtCu A-OSs/C	18	33.3
PtCu OSs/C	45	71.7

References:

[1] W.-D. Schneider, C. Laubschat, *Phys. Rev. B* **1981**, *23*, 997.

[2] C. Chen, Y. Kang, Z. Huo, Z. Zhu, W. Huang, H.L. Xin, J.D. Snyder, D. Li, J.A. Herron, M. Mavrikakis, M. Chi, K.L. More, Y. Li, N.M. Markovic, G.A. Somorjai, P. Yang, V.R. Stamenkovic, *Science* **2014**, *343*, 1339.

[3] S. Luo, M. Tang, P.K. Shen, S. Ye, *Adv. Mater.* **2017**, *29*.

[4] J. Ding, L. Bu, S. Guo, Z. Zhao, E. Zhu, Y. Huang, X. Huang, *Nano Lett.* **2016**, *16*, 2762.

[5] H.Y. Kim, T. Kwon, Y. Ha, M. Jun, H. Baik, H.Y. Jeong, H. Kim, K. Lee, S.H. Joo, *Nano Lett.* **2020**, *20*, 7413.

[6] S. Chen, J. Zhao, H. Su, H. Li, H. Wang, Z. Hu, J. Bao, J. Zeng, *J. Am. Chem. Soc.* **2021**, *143*, 496.

[7] T. Kwon, M. Jun, H.Y. Kim, A. Oh, J. Park, H. Baik, S.H. Joo, K. Lee, *Adv. Funct. Mater.* **2018**, *28*, 1706440.

[8] W. Ye, S. Chen, M. Ye, C. Ren, J. Ma, R. Long, C. Wang, J. Yang, L. Song, Y. Xiong, *Nano Energy* **2017**, *39*, 532.

[9] L. Huang, Z. Jiang, W. Gong, Z. Wang, P.K. Shen, *J. Power Sources* **2018**, *406*, 42.

[10] X. Sun, B. Huang, X. Cui, B. E, Y. Feng, X. Huang, *Chemcatchem* **2018**, *10*, 931.

[11] J. Park, H. Wang, M. Vara, Y. Xia, *Chemsuschem* **2016**, *9*, 2855.
